# Current Awareness on Comparative and Functional Genomics

**DOI:** 10.1002/cfg.422

**Published:** 2005


**Comparative and Functional Genomics**
*Comp Funct Genom* 2005; **6**: 326–333
Published online in Wiley InterScience (www.interscience.wiley.com). **DOI:** 10.1002cfg.422

### Current awareness on comparative and functional genomics

Current Awareness – Use the links online

### 1 Reviews & symposia

Abee T, Van Schaik W, Siezen RJ. 2004. Impact of genomics on microbial food safety (Review). *Trends Biotechnol* **22:** (12) 653.
Alaoui-Jamali MA, Dupre I, Qiang H. 2004. Prediction of drug sensitivity and drug resistance in cancer by transcriptional and proteomic profiling (Review). *Drug Dev Res* **7:** (4-5) 245.
Aldred S, Grant MM, Griffiths HR. 2004. The use of proteomics for the assessment of clinical samples in research (Review). *Clin Biochem* **37:** (11) 943.
Altman RB, Rubin DL, Klein TE. 2004. An "omics" view of drug development. *Drug Dev Res* **62:** (2) 81.
Arnett DK, Claas SA. 2004. Pharmacogenetics of antihypertensive treatment. *Drug Dev Res* **62:** (3) 191.
Barry R, Soloviev M. 2004. Quantitative protein profiling using antibody arrays (Review). *Proteomics* **4:** (12) 3717.
Bronstein P, Marrichi M, DeLisa MP. 2004. Dissecting the twin-arginine translocation pathway using genome-wide analysis (Review). *Res Microbiol* **155:** (10) 803.
Carlquist JF, Anderson JL. 2004. Pharmacogenomics in cardiovascular medicine. *Drug Dev Res* **62:** (3) 180.
Carucci DJ. 2004. *Plasmodium* post-genomics: An update. *Trends Parasitol* **20:** (12) 558.
Chin KV, Selyanayagam ZE, Vittal R, Kita T, Kudoh K, Yang CS, Wong YF, Cheung TH, Yeo W, Chung TKH *et al*. 2004. Application of expression genomics in drug development and genomic medicine. *Drug Dev Res* **62:** (2) 124.
Chiorino G, Acquadro F, Grand MM, Viscomi S, Segir R, Gasparini M, Dotto P. 2004. Interpretation of expression-profiling results obtained from different platforms and tissue sources: Examples using prostate cancer data (Review). *Eur J Cancer* **40:** (17) 2592.
Clarke PA, Te Poele R, Workman P. 2004. Gene expression microarray technologies in the development of new therapeutic agents (Review). *Eur J Cancer* **40:** (17) 2560.
Cooper JW, Wang YJ, Lee CS. 2004. Recent advances in capillary separations for proteomics. *Electrophoresis* **25:** (23-24) 3913.
Coppel RL, Roos DS, Bozdech Z. 2004. The genomics of malaria infection (Review). *Trends Parasitol* **20:** (12) 553.
Fleet JC. 2004. Genomic and proteomic approaches for probing the role of vitamin D in health. *Am J Clin Nutr* **80:** (6 Suppl) 1730S.
Flower DR, Attwood TK. 2004. Integrative bioinformatics for functional genome annotation: Trawling for G protein-coupled receptors. *Semin Cell Dev Biol* **15:** (6) 693.
Gabaldon T, Huynen MA. 2004. Shaping the mitochondrial proteome. *Biochim Biophys Acta* **1659:** (2-3) 212.
Galvin JE, Ginsberg SD. 2004. Expression profiling and pharmacotherapeutic development in the central nervous system (Review). *Alzheimer Dis Assoc Disord* **18:** (4) 264.
Goble C, Wroe C. 2004. The Montagues and the Capulets (Conference Paper). *Comp Funct Genom* **5:** (8) 623.
Gorg A, Weiss W, Dunn MJ. 2004. Current two-dimensional electrophoresis technology for proteomics (Review). *Proteomics* **4:** (12) 3665.
Haas DW. 2004. Pharmacogenomics of antiretroviral therapy. *Drug Dev Res* **62:** (3) 213.
Hakonarsson H, Stefansson K. 2004. Role of pharmacogenomics in drug development. *Drug Dev Res* **62:** (2) 86.
Handelsman J. 2004. Metagenomics: Application of genomics to uncultured microorganisms. *Microbiol Mol Biol Rev* **68:** (4) 669.
Hecker M, Volker U. 2004. Towards a comprehensive understanding of *Bacillus subtilis* cell physiology by physiological proteomics (Review). *Proteomics* **4:** (12) 3727.
Henke RT, Maitra A, Paik S, Wellstein A. 2005. Gene expression analysis in sections and tissue microarrays of archival tissues by mRNA *in situ* hybridization. *Histol Histopathol* **20:** (1) 225.
Lunshof J, De Wert G. 2004. Pharmacogenomics, drug development, and ethics: Some points to consider. *Drug Dev Res* **62:** (2) 112.
Matsumura H, Ito A, Saitoh H, Winter P, Kahl G, Reuter M, Kruger DH, Terauchi R. 2005. SuperSAGE. *Cell Microbiol* **7:** (1) 11.
Mayr M, Mayr U, Chung YL, Yin XK, Griffiths JR, Xul OB. 2004. Vascular proteomics: Linking proteomic and metabolomic changes (Review). *Proteomics* **4:** (12) 3751.
Mocellin S, Provenzano M, Rossi CR, Pilati P, Nitti D, Lise M. 2005. DNA array-based gene profiling - From surgical specimen to the molecular portrait of cancer (Review). *Ann Surg* **241:** (1) 16.
Mullighan CG, Bardy PG. 2004. Advances in the genomics of allogeneic haemopoietic stem cell transplantation. *Drug Dev Res* **62:** (3) 273.
Patterson LT, Potter SS. 2004. Profiling gene expression in kidney development (Review). *Nephron Exp Nephrol* **98:** (4) E109.
Ranganathan P, Eisen S. 2004. Pharmacogenomic approaches to therapies in rheumatic diseases. *Drug Dev Res* **62:** (3) 161.
Rapkiewicz AV, Espina V, Petricoin EF, Liotta LA. 2004. Biomarkers of ovarian tumours (Review). *Eur J Cancer* **40:** (17) 2604.
Reinders J, Lewandrowski U, Moebius J, Wagner Y, Sickmann A. 2004. Challenges in mass spectrometry-based proteomics (Review). *Proteomics* **4:** (12) 3686.

In order to keep subscribers up-to-date with the latest developments in their field, this current awareness service is provided by John Wiley & Sons and contains newly-published material on comparative and functional genomics. Each bibliography is divided into 16 sections. 1 Reviews & symposia; 2 General; 3 Large-scale sequencing and mapping; 4 Evolutionary genomics; 5 Comparative genomics; 6 Pathways, gene families and regulons; 7 Pharmacogenomics; 8 EST, cDNA and other clone resources; 9 Functional genomics; 10 Transcriptomics; 11 Proteomics; 12 Protein structural genomics; 13 Metabolomics; 14 Genomic approaches to development; 15 Technological advances; 16 Bioinformatics. Within each section, articles are listed in alphabetical order with respect to author. If, in the preceding period, no publications are located relevant to any one of these headings, that section will be omitted.

Copyright © 2005 John Wiley & Sons, Ltd.

Robson B. 2004. The dragon on the gold: Myths and realities for data mining in biomedicine and biotechnology using digital and molecular libraries (Review). *J Proteome Res* **3:** (6) 1113.

Rohde LA, Zeni C, Polanczyk G, Hutz MH. 2004. New insights on attention-deficit/hyperactivity disorder pharmacogenomics. *Drug Dev Res* **62:** (3) 172.

Rosmond R. 2004. Pharmacogenomic approaches in antidiabetic drug development. *Drug Dev Res* **62:** (3) 207.

Sansone SA, Morrison N, Rocca-Serra P, Fostel J. 2004. Standardization initiatives in the (eco)toxicogenomics domain: A review (Conference Paper). *Comp Funct Genom* **5:** (8) 633.

Sausville EA, Holbeck SL. 2004. Transcription profiling of gene expression in drug discovery and development: The NO experience (Review). *Eur J Cancer* **40:** (17) 2544.

Sinden RE. 2004. A proteomic analysis of malaria biology: Integration of old literature and new technologies (Review). *Int J Parasitol* **34:** (13-14) 1441.

Smith PM, Ross GF, Taylor RW, Turnbull DA, Lightowlers RN. 2004. Strategies for treating disorders of the mitochondrial genome. *Biochim Biophys Acta* **1659:** (2-3) 232.

Stasyk T, Huber LA. 2004. Zooming in: Fractionation strategies in proteomics (Review). *Proteomics* **4:** (12) 3704.

Taramelli R, Acquati F. 2004. The human genome project and the discovery of genetic determinants of cancer susceptibility (Review). *Eur J Cancer* **40:** (17) 2537.

Wulfkuhle J, Espina V, Liotta L, Petricoin E. 2004. Genomic and proteomic technologies for individualisation and improvement of cancer treatment (Review). *Eur J Cancer* **40:** (17) 2623.

Zeng CY, Sanada H, Watanabe H, Eisner GM, Felder RA, Jose PA. 2004. Functional genomics of the dopaminergic system in hypertension (Review). *Physiol Genomics* **19:** (3) 233.

### 3 Large-scale sequencing and mapping

Bolotin A, Quinquis B, Renault P, Sorokin A, Ehrlich SD, Kulakauskas S, Lapidus A, Goltsman E, Mazur M, Pusch GD *et al*. 2004. Complete sequence and comparative genome analysis of the dairy bacterium *Streptococcus thermophilus*. *Nat Biotechnol* **22:** (12) 1554.

Ivshina AV, Vodeiko GM, Kuznetsov VA, Volokhov D, Taffs R, Chizhikov VI, Levandowski RA, Chumakov KM. 2004. Mapping of genomic segments of influenza B virus strains by an oligonucleotide microarray method. *J Clin Microbiol* **42:** (12) 5793.

Rabus R, Kube M, Heider J, Beck A, Heitmann K, Widdel F, Reinhardt R. 2005. The genome sequence of an anaerobic aromatic-degrading denitrifying bacterium, strain EbN1. *Arch Microbiol* **183:** (1) 27.

Seshadri R, Adrian L, Fouts DE, Eisen JA, Phillippy AM, Methe BA, Ward NL, Nelson WC, Deboy RT, Khouri HM *et al*. 2005. Genome sequence of the PCE-dechlorinating bacterium *Dehalococcoides ethenogenes*. *Science* **307:** (5706) 105.

### 4 Evolutionary genomics

Blanchette M, Green ED, Miller W, Haussler D. 2004. Reconstructing large regions of an ancestral mammalian genome *in silico*. *Genome Res* **14:** (12) 2412.

Brinkmann H, Denk A, Zitzler J, Joss JJ, Meyer A. 2004. Complete mitochondrial genome sequences of the South American and the Australian lungfish: Testing of the phylogenetic performance of mitochondrial data sets for phylogenetic problems in tetrapod relationships. *J Mol Evol* **59:** (6) 834.

Fadiel A, Lithwick S, Naftolin F. 2005. The influence of environmental adaptation on bacterial genome structure. *Lett Appl Microbiol* **40:** (1) 12.

Hughes AL, Friedman R. 2004. Shedding genomic ballast: Extensive parallel loss of ancestral gene families in animals. *J Mol Evol* **59:** (6) 827.

Noonan JP, Grimwood J, Danke J, Schmutz J, Dickson M, Amemiya CT, Myers RM. 2004. Coelacanth genome sequence reveals the evolutionary history of vertebrate genes. *Genome Res* **14:** (12) 2397.

Swanson WJ, Wong A, Wolfner MF, Aquadro CF. 2004. Evolutionary expressed sequence tag analysis of *Drosophila* female reproductive tracts identifies genes subjected to positive selection. *Genetics* **168:** (3) 1457.

Takami H, Takaki Y, Chee GJ, Nishi S, Shimamura S, Suzuki H, Matsui S, Uchiyama I. 2004. Thermoadaptation trait revealed by the genome sequence of thermophilic *Geobacillus kaustophilus*. *Nucleic Acids Res* **32:** (21) 6292.

Thomarat F, Vivares CP, Gouy M. 2004. Phylogenetic analysis of the complete genome sequence of *Encephalitozoon cuniculi* supports the fungal origin of microsporidia and reveals a high frequency of fast-evolving genes. *J Mol Evol* **59:** (6) 780.

### 5 Comparative genomics

Baptista CS, Vencio RZN, Abdala S, Valadares MP, Martins C, Pereira CAD, Zingales B. 2004. DNA microarrays for comparative genomics and analysis of gene expression in *Trypanosoma cruzi*. *Mol Biochem Parasitol* **138:** (2) 183.

Boffelli D, Weer CV, Weng L, Lewis KD, Shoukry MI, Pachter L, Keys DN, Rubin EM. 2004. Intraspecies sequence comparison for annotating genomes. *Genome Res* **14:** (12) 2406.

Chibani-Chennoufi S, Canchaya C, Bruttin A, Brussow H. 2004. Comparative genomics of the T4-like *Escherichia coli* phage JS98: Implications for the evolution of T4 phages. *J Bacteriol* **186:** (24) 8276.

Rodionov DA, Gelfand MS, Hugouvieux-Cotte-Pattat N. 2004. Comparative genomics of the KdgR regulon in *Erwinia chrysanthemi* 3937 and other gamma-proteobacteria. *Microbiology* **150:** (11) 3571.

### 7 Pharmacogenomics

Adur J, Uchide T, Takizawa S, Quan JX, Saida K. 2004. Real-time polymerase chain reaction quantification of gene expression levels of murine endothelin-A and endothelin-B receptors: Gene expression profiles by the standard curve method. *J Cardiovasc Pharmacol* **44:** (Suppl 1) S321.

Aggarwal A, Leong SH, Lee C Kon OL, Tan P. 2005. Wavelet transformations of tumor expression profiles reveals a pervasive genome-wide imprinting of aneuploidy on the cancer transcriptome. *Cancer Res* **65:** (1) 186.

Ami Y, Shimazu T, Akaza H, Uematsu N, Yano Y, Tsujimoto G, Uchida K. 2005. Gene expression profiles correlate with the morphology and metastasis characteristics of renal cell carcinoma cells. *Oncol Rep* **13:** (1) 75.

Anderson SP, Howroyd P, Liu J, Qian X, Bahnemann R, Swanson C, Kwak MK, Kensler TW, Corton JC. 2004. The transcriptional response to a peroxisome proliferator-activated receptor α agonist includes increased expression of proteome maintenance genes. *J Biol Chem* **279:** (50) 52390.

Armendariz AD, Gonzalez M, Loguinov AV, Vulpe CD. 2004. Gene expression profiling in chronic copper overload reveals upregulation of Prnp and App. *Physiol Genomics* **20:** (1) 45.

Barad O, Meiri E, Avniel A, Aharonov R, Barzilai A, Bentwich I, Einav U, Glad S, Hurban P, Karov Y *et al*. 2004. MicroRNA expression detected by oligonucleotide microarrays: System establishment and expression profiling in human tissues. *Genome Res* **14:** (12) 2486.

Barber DS, LoPachin RM. 2004. Proteomic analysis of acrylamide-protein adduct formation in rat brain synaptosomes. *Toxicol Appl Pharmacol* **201:** (2) 120.

Barglow KT, Cravatt BF. 2004. Discovering disease-associated enzymes by proteome reactivity profiling. *Chem Biol* **11:** (11) 1523.

Basso M, Giraudo S, Corpillo D, Bergamasco B, Lopiano L, Fasano M. 2004. Proteome analysis of human substantia nigra in Parkinson's disease. *Proteomics* **4:** (12) 3943.

Bibikova M, Yeakley JM, Chudin E, Jing C, Wickham E, Wang-Rodriguez J, Fan JB. 2004. Gene expression profiles in formalin-fixed, paraffin-embedded tissues obtained with a novel assay for microarray analysis. *Clin Chem* **50:** (12) 2384.

Booth S, Bowman C, Baumgartner R, Dolenko B, Sorensen G, Robertson C, Coulthart M, Phillipson C, Somorjai R. 2004. Molecular classification of scrapie strains in mice using gene expression profiling. *Biochem Biophys Res Commun* **325:** (4) 1339.

Bouwman F, Renes J, Mariman E. 2004. A combination of protein profiling and isotopomer analysis using matrix-assisted laser desorption/ionization-time of flight mass spectrometry reveals an active metabolism of the extracellular matrix of 3T3-L1 adipocytes. *Proteomics* **4:** (12) 3855.

Copyright © 2005 John Wiley & Sons, Ltd. 

Carlen LM, Sanchez F, Bergman AC, Becker S, Hirschberg D, Franzen B, Coffey J, Jornvall H, Auer G, Alaiya AA *et al*. 2005. Proteome analysis of skin distinguishes acute guttate from chronic plaque psoriasis. *J Investig Dermatol* **124:** (1) 63.

Chen JH, Chang YW, Yao CW, Chiueh TS, Huang SC, Chien KY, Chen A, Chang FY, Wong CH, Chen YJ. 2004. Plasma proteome of severe acute respiratory syndrome analyzed by two-dimensional gel electrophoresis and mass spectrometry. *Proc Natl Acad Sci U S A* **101:** (49) 17039.

Chou KC. 2004. Molecular therapeutic target for type 2 diabetes. *J Proteome Res* **3:** (6) 1284.

Chromy BA, Gonzales AD, Perkins J, Choi MW, Corzett MH, Chang BC, Corzett CH, McCutchen-Maloney SL. 2004. Proteomic analysis of human serum by two-dimensional differential gel electrophoresis after depletion of high-abundant proteins. *J Proteome Res* **3:** (6) 1120.

Colland F, Daviet L. 2004. Integrating a functional proteomic approach into the target discovery process. *Biochimie* **86:** (9-10) 625.

Craven RA, Stanley AJ, Hanrahan S, Totty N, Jackson DP, Popescu R, Taylor A, Frey J, Selby PJ, Patel PM *et al*. 2004. Identification of proteins regulated by interferon-α in resistant and sensitive malignant melanoma cell lines. *Proteomics* **4:** (12) 3998.

Da Silva R, Lucchinetti E, Pasch T, Schaub MC, Zaugg M. 2004. Ischemic but not pharmacological preconditioning elicits a gene expression profile similar to unprotected myocardium. *Physiol Genomics* **20:** (1) 117.

Davezac N, Tondelier D, Lipecka J, Fanen P, Demaugre F, Debski J, Dadlez M, Schrattenholz A, Cahill MA, Edelman A. 2004. Global proteomic approach unmasks involvement of keratins 8 and 18 in the delivery of cystic fibrosis transmembrane conductance regulator (CFTR)ΔF508-CFTR to the plasma membrane. *Proteomics* **4:** (12) 3833.

Deshane J, Chaves L, Sarikonda KV, Isbell S, Wilson L, Kirk M, Grubbs C, Barnes S, Meleth S, Kim H. 2004. Proteomics analysis of rat brain protein modulations by grape seed extract. *J Agric Food Chem* **52:** (26) 7872.

Diehl D, Lahm H, Wolf E, Bauersachs S. 2005. Transcriptome analysis of a human colorectal cancer cell line shows molecular targets of insulin-like growth factor-binding protein-4 overexpression. *Int J Cancer* **113:** (4) 588.

Dos Santos CC, Han B, Andrade CF, Bai X, Uhlig S, Hubmayr R, Tsang M, Lodyga M, Keshavjee S *et al*. 2004. DNA microarray analysis of gene expression in alveolar epithelial cells in response to TNFα, LPS, and cyclic stretch. *Physiol Genomics* **19:** (3) 331.

Ebert MPA, Meuer J, Wiemer JC, Schulz HU, Reymond MA, Traugott U, Malfertheiner P, Rocken C. 2004. Identification of gastric cancer patients by serum protein profiling. *J Proteome Res* **3:** (6) 1261.

Eisenberger S, Hoppe G, Pyerin W, Ackermann K. 2004. High-quality RNA preparation for transcript profiling of osteocytes from native human bone microdissections. *Anal Biochem* **335:** (2) 260.

Fahey ME, Mills W, Higgins DG, Moore T. 2004. Maternally and paternally silenced imprinted genes differ in their intron content. *Comp Funct Genom* **5:** (8) 572.

Feezor RJ, Baker HV, Mindrinos M, Hayden D, Tannahill CL, Brownstein BH, Fay A, MacMillan S, Laramie J, Xiao WZ *et al*. 2004. Whole blood and leukocyte RNA isolation for gene expression analyses. *Physiol Genomics* **19:** (3) 247.

Finley DJ, Zhu BX, Fahey TJ. 2004. Molecular analysis of Hurthle cell neoplasms by gene profiling. *Surgery* **136:** (6) 1160.

Fukushima N, Sato N, Prasad N, Leach SD, Hruban RH, Goggins M. 2004. Characterization of gene expression in mucinous cystic neoplasms of the pancreas using oligonucleotide microarrays. *Oncogene* **23:** (56) 9042.

Fung KYC, Morris C, Sathe S, Sack R, Duncan MW. 2004. Characterization of the *in vivo* forms of lacrimal-specific proline-rich proteins in human tear fluid. *Proteomics* **4:** (12) 3953.

Giannone S, Strippoli P, Vitale L, Casadei R, Canaider S, Lenzi L, D'Addabbo R, Frabetti F, Facchin F, Farina A *et al*. 2004. Gene expression profile analysis in human T lymphocytes from patients with Down syndrome. *Ann Hum Genet* **68:** (6) 546.

Glas AM, Kersten MJ, Delahaye LJMJ, Witteveen AT, Kibbelaar RE, Velds A, Wessels LFA, Joosten P, Kerkhoven RM, Bernards R *et al*. 2005. Gene expression profiling in follicular lymphoma to assess clinical aggressiveness and to guide the choice of treatment. *Blood* **105:** (1) 301.

Golpon HA, Coldren CD, Zamora MR, Cosgrove GP, Moore MD, Tuder RM, Geraci MW, Voelkel NF. 2004. Emphysema lung tissue gene expression profiling. *Am J Respir Cell Mol Biol* **31:** (6) 595.

Gu S, Du YC, Chen J, Liu ZH, Bradbury EM, Hu CAA, Chen X. 2004. Large-scale quantitative proteomic study of PUMA-induced apoptosis using two-dimensional liquid chromatography-mass spectrometry coupled with amino acid-coded mass tagging. *J Proteome Res* **3:** (6) 1191.

Gwadry FG, Sequeira A, Hoke G, Ffrench-Mullen JMH, Turecki G. 2005. Molecular characterization of suicide by microarray analysis. *Am J Med Genet* **133C:** (1) 48.

Huber A, Hudelist G, Czerwenka K, Husslein P, Kubista E, Singer CF. 2005. Gene expression profiling of cervical tissue during physiological cervical effacement. *Obstet Gynecol* **105:** (1) 91.

Inoue Y, Shirane M, Miki C, Hiro J, Tanaka K, Kobayashi M, Mori K, Yanagi H, Kusunoki M. 2004. Gene expression profiles of colorectal carcinoma in response to neo-adjuvant chemotherapy. *Int J Oncol* **25:** (6) 1641.

Iwaki H, Kageyama S, Isono T, Wakabayashi Y, Okada Y, Yoshimura K, Terai A, Arai Y, Iwamura H, Kawakita M *et al*. 2004. Diagnostic potential in bladder cancer of a panel of tumor markers (calreticulin, γ-synuclein, and catechol-*o*-methyltransferase) identified by proteomic analysis. *Cancer Sci* **95:** (12) 955.

Jeyaseelan S, Chu HW, Young SK, Worthen GS. 2004. Transcriptional profiling of lipopolysaccharide-induced acute lung injury. *Infect Immun* **72:** (12) 7247.

Kamat AA, Younes PS, Sayeeduddin M, Wheeler TM, Simpson JL, Agoulnik AI. 2004. Protein expression profiling of endometriosis: Validation of 2-mm tissue microarrays (Letter). *Fertil Steril* **82:** (6) 1681.

Kang XX, Xu Y, Wu XY, Liang Y, Wang C, Guo JH, Wang YJ, Chen MH, Wu D, Wang YC *et al*. 2005. Proteomic fingerprints for potential application to early diagnosis of severe acute respiratory syndrome. *Clin Chem* **51:** (1) 56.

Kanski J, Behring A, Pelling J, Schoneich C. 2005. Proteomic identification of 3-nitrotyrosine-containing rat cardiac proteins: Effects of biological aging. *Am J Physiol* **288:** (1) H371.

Kitamura H, Nakagawa T, Takayama M, Kimura Y, Hijika A, Ohara O. 2004. Post-transcriptional effects of phorbol 12-myristate 13-acetate on transcriptome of U937 cells. *FEBS Lett* **578:** (1-2) 180.

Koenig-Hoffmann K, Bonin-Debs AL, Boche I, Gawin B, Gnirke A, Hergersberg C, Madeo F, Kazinski M, Klein M, Korherr C *et al*. 2005. High throughput functional genomics: Identification of novel genes with tumor suppressor phenotypes. *Int J Cancer* **113:** (3) 434.

Koike E, Hirano S, Furuyama A, Kobayashi T. 2004. cDNA microarray analysis of rat alveolar epithelial cells following exposure to organic extract of diesel exhaust particles. *Toxicol Appl Pharmacol* **201:** (2) 178.

Kumar RN, Radhakrishnan R, Ha JH, Dhanasekaran N. 2004. Proteome analysis of NIH3T3 cells transformed by activated $G\alpha_{12}$: Regulation of leukemia-associated protein SET. *J Proteome Res* **3:** (6) 1177.

Lee JD, Yun MJ, Lee JM, Choi YJ, Choi YH, Kim JS, Kim SJ, Kim KS, Yang WI, Park YN *et al*. 2004. Analysis of gene expression profiles of hepatocellular carcinomas with regard to $^{18}$F-fluorodeoxyglucose uptake pattern on positron emission tomography. *Eur J Nucl Med Mol Imaging* **31:** (12) 1621.

Lee JS, Chu IS, Mikaelyan A, Calvisi DF, Heo J, Reddy JK, Thorgeirsson SS. 2004. Application of comparative functional genomics to identify best-fit mouse models to study human cancer. *Nat Genet* **36:** (12) 1306.

Li MD, Kane JK, Ju W, Ma JZ. 2004. Time-dependent changes in transcriptional profiles within five rat brain regions in response to nicotine treatment. *Mol Brain Res* **132:** (2) 168.

Liu AY, Zhang H, Sorensen CM, Diamond DL. 2005. Analysis of prostate cancer by proteomics using tissue specimens. *J Urol* **173:** (1) 73.

Low TY, Leow CK, Salto-Tellez M, Chung MCM. 2004. A proteomic analysis of thioacetamide-induced hepatotoxicity and cirrhosis in rat livers. *Proteomics* **4:** (12) 3960.

Lu ZH, Hu LP, Evers S, Chen J, Shen Y. 2004. Differential expression profiling of human pancreatic adenocarcinoma and healthy pancreatic tissue. *Proteomics* **4:** (12) 3975.

Malyarenko DI, Cooke WE, Adam BL, Malik G, Chen HJ, Tracy ER, Trosset MW, Sasinowski M, Semmes OJ, Manos DM. 2005. Enhancement of sensitivity and resolution of surface-enhanced, laser desorption/ionization time-of-flight mass spectrometric records for se-

Copyright © 2005 John Wiley & Sons, Ltd. 

rum peptides using time-series analysis techniques. *Clin Chem* **51:** (1) 65.

McIntyre A, Summersgill B, Jafer O, Rodriguez S, Zafarana G, Oosterhuis JW, Gillis AJM, Looijenga L, Cooper C, Huddart R *et al*. 2004. Defining minimum genomic regions of imbalance involved in testicular germ cell tumors of adolescents and adults through genome wide microarray analysis of cDNA clones. *Oncogene* **23:** (56) 9142.

Mears R, Craven RA, Hanrahan S, Totty N, Upton C, Young SL, Patel P, Selby PJ, Banks RE. 2004. Proteomic analysis of melanoma-derived exosomes by two-dimensional polyacrylamide gel electrophoresis and mass spectrometry. *Proteomics* **4:** (12) 4019.

Morris DL, Sutton JN, Harper RG, Timperman AT. 2004. Reversed-phase HPLC separation of human serum employing a novel saw-tooth gradient: Toward multidimensional proteome analysis. *J Proteome Res* **3:** (6) 1149.

Murakawa K, Tada M, Takada M, Tamoto E, Shindoh G, Teramoto K, Matsunaga A, Komuro K, Kanai M, Kawakami A *et al*. 2004. Prediction of lymph node metastasis and perineural invasion of biliary tract cancer by selected features from cDNA array data. *J Surg Res* **122:** (2) 184.

Murray J, Yonally S, Aggeler R, Marusich MF, Capaldi RA. 2004. Focused proteomics: Towards a high throughput monoclonal antibody-based resolution of proteins for diagnosis of mitochondrial diseases. *Biochim Biophys Acta* **1659:** (2-3) 206.

O'Riordan E, Orlova TN, Mei JF, Butt K, Chander PM, Rahman S, Mya M, Hu R, Momin J, Eng EW *et al*. 2004. Bioinformatic analysis of the urine proteome of acute allograft rejection. *J Am Soc Nephrol* **15:** (12) 3240.

Ohali A, Avigad S, Zaizov R, Ophir R, Horn-Saban S, Cohen IJ, Meller I, Kollender Y, Issakov J, Yaniv I. 2004. Prediction of high risk Ewing's sarcoma by gene expression profiling. *Oncogene* **23:** (55) 8997.

Okamura M, Sumida K, Muto T, Kashida Y, Machida N, Watanabe T, Mitsumori K. 2004. Analysis of gene expression profiles of forestomach tumors in rasH2 mice initiated with *N*-ethyl-*N*-nitrosourea. *Arch Toxicol* **78:** (12) 688.

Ong ES, Len SM, Lee ACH. 2005. Differential protein expression of the inhibitory effects of a standardized extract from *Scutellariae* Radix in liver cancer cell lines using liquid chromatography and tandem mass spectrometry. *J Agric Food Chem* **53:** (1) 8.

Palotas M, Palotas A, Puskas LG, Kitajka K, Pakaski M, Janka Z, Molnar J, Penke B, Kalman J. 2004. Gene expression profile analysis of the rat cortex following treatment with imipramine and citalopram. *Int J Neuropsychopharmacol* **7:** (4) 401.

Parkar MH, Tonetti M. 2004. Gene expression profiles of periodontol ligament cells treated with enamel matrix proteins *in vitro*: Analysis using cDNA arrays. *J Periodontol* **75:** (11) 1539.

Planaguma J, Diaz-Fuertes M, Gil-Moreno A, Abal M, Monge M, Garcia A, Baro T, Thomson TM, Xercavins J, Alameda F *et al*. 2004. A differential gene expression profile reveals overexpression of *RUNX1/AML1* in invasive endometrioid carcinoma. *Cancer Res* **64:** (24) 8846.

Rajput A, Singh B. 2004. Gene expression profiling in type 1 diabetes prone NOD mice immunized with a disease protective autoantigenic peptide. *J Autoimmun* **23:** (4) 311.

Ramstrom M, Ivonin I, Johansson A, Askmark H, Markides KE, Zubarev R, Hakansson P, Aquilonius SM, Bergquist J. 2004. Cerebrospinal fluid protein patterns in neurodegenerative disease revealed by liquid chromatography-Fourier transform ion cyclotron resonance mass spectrometry. *Proteomics* **4:** (12) 4010.

Reyzer ML, Caldwell RL, Dugger TC, Forbes JT, Ritter CA, Guix M, Arteaga CL, Caprioli RM. 2004. Early changes in protein expression detected by mass spectrometry predict tumor response to molecular therapeutics. *Cancer Res* **64:** (24) 9093.

Semmes OJ, Feng Z, Adam BL, Banez LL, Bigbee WL, Campos D, Cazares LH, Chan DW, Grizzle WE, Izbicka E *et al*. 2005. Evaluation of serum protein profiling by surface-enhanced laser desorption/ionization time-of-flight mass spectrometry for the detection of prostate cancer: I. Assessment of platform reproducibility. *Clin Chem* **51:** (1) 102.

Spijker S, Van de Leemput JCH, Hoekstra C, Boomsma DI, Smit AB. 2004. Profiling gene expression in whole blood samples following an *in-vitro* challenge. *Twin Res* **7:** (6) 564.

Spin JM, Nallamshetty S, Tabibiazar R, Ashley EA, King JY, Chen M, Tsao PS, Quertermous T. 2004. Transcriptional profiling of *in vitro* smooth muscle cell differentiation identifies specific patterns of gene and pathway activation. *Physiol Genomics* **19:** (3) 292.

Spira A, Beane J, Pinto-Plata V, Kadar A, Liu G, Shah V, Celli B, Brody JS. 2004. Gene expression profiling of human lung tissue from smokers with severe emphysema. *Am J Respir Cell Mol Biol* **31:** (6) 601.

Steffensen KR, Neo SY, Stulnig TM, Vega VB, Rahman SS, Schuster GU, Gustafsson JA, Liu ET. 2004. Genome-wide expression profiling: A panel of mouse tissues discloses novel biological functions of liver X receptors in adrenals. *J Mol Endocrinol* **33:** (3) 609.

Takahashi H, Masuda K, Ando T, Kobayashi T, Honda H. 2004. Prognostic predictor with multiple fuzzy neural models using expression profiles from DNA microarray for metastases of breast cancer. *J Biosci Bioeng* **98:** (3) 193.

Taulan M, Paquet F, Maubert C, Delissen O, Demaille J, Romey MC. 2004. Renal toxicogenomic response to chronic uranyl nitrate insult in mice. *Environ Health Perspect* **112:** (16) 1628.

Tsuda H, Birrer MJ, Ito YM, Ohashi Y, Lin M, Lee C, Wong WH, Rao PH, Lau CC, Berkowitz RS *et al*. 2004. Identification of DNA copy number changes in microdissected serous ovarian cancer tissue using a cDNA microarray platform. *Cancer Genet Cytogenet* **155:** (2) 97.

Vey N, Mozziconacci MJ, Groulet-Martinec A, Debono S, Finetti P, Carbuccia N, Beillard E, Devilard E, Arnoulet C, Coso D *et al*. 2004. Identification of new classes among acute myelogenous leukaemias with normal karyotype using gene expression profiling. *Oncogene* **23:** (58) 9381.

Wagner RA, Tabibiazar R, Powers J, Bernstein D, Quertermous T. 2004. Genome-wide expression profiling of a cardiac pressure overload model identifies major metabolic and signaling pathway responses. *J Mol Cell Cardiol* **37:** (6) 1159.

Xiang R, Shi Y, Dillon DA, Negin B, Horvath C, Wilkins JA. 2004. 2D LC/MS analysis of membrane proteins from breast cancer cell lines MCF7 and BT474. *J Proteome Res* **3:** (6) 1278.

Yang T, Lu AG, Ran RQ, Aronow BJ, Schorry EK, Hopkin RJ, Gilbert DL, Glauser TA, Hershey AD, Richtand NW *et al*. 2004. Human blood genomics: Distinct profiles for gender, age and neurofibromatosis type 1. *Mol Brain Res* **132:** (2) 155.

Yeom M, Shim I, Lee HJ, Hahm DH. 2005. Proteomic analysis of nicotine-associated protein expression in the striatum of repeated nicotine-treated rats. *Biochem Biophys Res Commun* **326:** (2) 321.

Yim EK, Lee KH, Bae JS, Namkoong SE, Um SJ, Park JS. 2004. Proteomic analysis of antiproliferative effects by treatment of 5-fluorouracil in cervical cancer cells. *DNA Cell Biol* **23:** (11) 769.

Yim EK, Meoyng J, Namkoong SE, Um SJ, Park JS. 2004. Genomic and proteomic expression patterns in HPV-16 E6 gene transfected stable human carcinoma cell lines. *DNA Cell Biol* **23:** (12) 826.

Yip TTC, Chan JWM, Cho WCS, Yip TT, Wang Z, Kwan TL, Law SCK, Tsang DNC, Chan JKC, Lee KC *et al*. 2005. Protein chip array profiling analysis in patients with severe acute respiratory syndrome identified serum amyloid a protein as a biomarker potentially useful in monitoring the extent of pneumonia. *Clin Chem* **51:** (1) 47.

You L, Bartolucci EJ. 2004. Gene expression profiles in mammary gland of male rats treated with genistein and methoxychlor. *Environ Toxicol Pharmacol* **18:** (2) 161.

Zineh I, Gerhard T, Aquilante CL, Beitelshees AL, Beasley BN, Hartzema AG. 2004. Availability of pharmacogenomics-based prescribing information in drug package inserts for currently approved drugs. *Pharmacogenomics J* **4:** (6) 354.

Zou MJ, Famulski KS, Parhar RS, Baitei E, Al-Mohanna FA, Farid NR, Shi YF. 2004. Microarray analysis of metastasis-associated gene expression profiling in a murine model of thyroid carcinoma pulmonary metastasis: Identification of S100A4 (Mts1) gene overexpression as a poor prognostic marker for thyroid carcinoma. *J Clin Endocrinol Metab* **89:** (12) 6146.

### 8 EST, cDNA and other clone resources

Gilchrist MJ, Zorn AM, Voigt J, Smith JC, Papalopulu N, Amaya E. 2004. Defining a large set of full-length clones from a *Xenopus tropicalis* EST project. *Dev Biol* **271:** (2) 498.

Howson R, Huh WK, Ghaemmaghami S, Falvo JV, Bower K, Belle A, Dephoure N, Wykoff DD, Weissman JS, O'Shea EK. 2005. Construction, verification and experimental use of two epitope-tagged collections of budding yeast strains. *Comp Funct Genom* **6:** (1-2) 2.

Ogihara Y, Mochida K, Kawaura K, Murai K, Seki M, Kamiya A,

Copyright © 2005 John Wiley & Sons, Ltd.

Shinozaki K, Carninci P, Hayashizaki Y, Shin-I T *et al*. 2004. Construction of a full-length cDNA library from young spikelets of hexaploid wheat and its characterization by large-scale sequencing of expressed sequence tags. *Genes Genet Syst* **79:** (4) 227.
Suzuki T, Ota T, Fujiyama A, Kasahara M. 2004. Construction of a bacterial artificial chromosome library from the inshore hagfish, *Eptatretus burgeri*: A resource for the analysis of the agnathan genome. *Genes Genet Syst* **79:** (4) 251.
Wang LL, Ma L, Leng WC, Yang J, Zhu JP, Dong J, Xue Y, Wan Z, Li RY, Jin Q. 2004. Analysis of part of the *Trichophyton rubrum* ESTs. *Sci China C Life Sci* **47:** (5) 389.

### 9 Functional genomics

Bettencourt-Dias M, Giet R, Sinka R, Mazumdar A, Lock WG, Balloux F, Zafiropoulos PJ, Yamaguchi S, Winter S, Carthew RW *et al*. 2004. Genome-wide survey of protein kinases required for cell cycle progression. *Nature* **432:** (7020) 980.
Doerks T, Von Mering C, Bork P. 2004. Functional clues for hypothetical proteins based on genomic context analysis in prokaryotes. *Nucleic Acids Res* **32:** (21) 6321.
Oakley TH, Gu ZL, Abouheif E, Patel NH, Li WH. 2005. Comparative methods for the analysis of gene-expression evolution: An example using yeast functional genomic data. *Mol Biol Evol* **22:** (1) 40.
Richman A, Swanson A, Humphrey T, Chapman R, McGarvey B, Pocs R, Brandle J. 2005. Functional genomics uncovers three glucosyltransferases involved in the synthesis of the major sweet glucosides of *Stevia rebaudiana*. *Plant J* **41:** (1) 56.
Von Rozycki T, Nies DH, Saier MH. 2005. Genomic analyses of transport proteins in *Ralstonia metallidurans*. *Comp Funct Genom* **6:** (1-2) 17.
Wiseman A. 2005. Avoidance of oxidative-stress perturbation in yeast bioprocesses by proteomic and genomic biostrategies? *Lett Appl Microbiol* **40:** (1) 37.
Xu ZW, Escamilla-Trevino LL, Zeng LH, Lalgondar M, Bevan DR, Winkel BSJ, Mohamed A, Cheng CL, Shih MC, Poulton JE *et al*. 2004. Functional genomic analysis of *Arabidopsis thaliana* glycoside hydrolase family 1. *Plant Mol Biol* **55:** (3) 343.

### I0 Transcriptomics

Altenbach SB, Kothari KM. 2004. Transcript profiles of genes expressed in endosperm tissue are altered by high temperature during wheat grain development. *J Cereal Sci* **40:** (2) 115.
Bailey MJ, Beremand PD, Hammer R, Reidel E, Thomas TK, Cassone VM. 2004. Transcriptional profiling of circadian patterns of mRNA expression in the chick retina. *J Biol Chem* **279:** (50) 52247.
Bertone P, Stolc V, Royce TE, Rozowsky JS, Urban AE, Zhu XW, Rinn JL, Tongprasit W, Samanta M, Weissman S *et al*. 2004. Global identification of human transcribed sequences with genome tiling arrays. *Science* **306:** (5705) 2242.
Davalos M, Fourment J, Lucas A, Berges H, Kahn D. 2004. Nitrogen regulation in *Sinorhizobium meliloti* probed with whole genome arrays. *FEMS Microbiol Lett* **241:** (1) 33.
Gracey AY, Fraser EJ, Li WZ, Fang YX, Taylor RR, Rogers J, Brass A, Cossins AR. 2004. Coping with cold: An integrative, multitissue analysis of the transcriptome of a poikilothermic vertebrate. *Proc Natl Acad Sci U S A* **101:** (48) 16970.
Hornbaek T, Jakobsen M, Dynesen J, Nielsen AK. 2004. Global transcription profiles and intracellular pH regulation measured in *Bacillus licheniformis* upon external pH upshifts. *Arch Microbiol* **182:** (6) 467.
Ko JH, Han KH. 2004. *Arabidopsis* whole-transcriptome profiling defines the features of coordinated regulations that occur during secondary growth. *Plant Mol Biol* **55:** (3) 433.
Lehnert SA, Wang YH, Byrne KA. 2004. Development and application of a bovine cDNA microarray for expression profiling of muscle and adipose tissue. *Aust J Exp Agric* **44:** (11) 1127.
Marathe R, Guan Z, Anandalakshmi R, Zhao HY, Dinesh-Kumar SP. 2004. Study of *Arabidopsis thaliana* resistome in response to cucumber mosaic virus infection using whole genome microarray. *Plant Mol Biol* **55:** (4) 501.
Pan D, He NH, Yang ZY, Liu HP, Xu X. 2005. Differential gene expression profile in hepatopancreas of WSSV-resistant shrimp (*Penaeus japonicus*) by suppression subtractive hybridization. *Dev Comp Immunol* **29:** (2) 103.
Parkinson J, Mitreva M, Whitton C, Thomson M, Daub J, Martin J, Schmid R, Hall N, Barrell B, Waterson RH *et al*. 2004. A transcriptomic analysis of the phylum Nematoda. *Nat Genet* **36:** (12) 1259.
Rong YQ, Wang TY, Morgan JI. 2004. Identification of candidate Purkinje cell-specific markers by gene expression profiling in wild-type and pcd$^{3J}$ mice. *Mol Brain Res* **132:** (2) 128.
Tai SL, Boer VM, Daran-Lapujade P, Walsh MC, De Winde JH, Daran JM, Pronk JT. 2005. Two-dimensional transcriptome analysis in chemostat cultures - Combinatorial effects of oxygen availability and macronutrient limitation in *Saccharomyces cerevisiae*. *J Biol Chem* **280:** (1) 437.
Toyoda M, Cho T, Kaminishi H, Sudoh M, Chibana H. 2004. Transcriptional profiling of the early stages of germination of *Candida albicans* by real-time RT-PCR. *FEMS Yeast Res* **5:** (3) 287.
Wagner VE, Gillis RJ, Iglewski BH. 2004. Transcriptome analysis of quorum-sensing regulation and virulence factor expression in *Pseudomonas aeruginosa*. *Vaccine* **22:** (Suppl 1) S15.
Wan XF, Verberkmoes NC, McCue LA, Stanek D, Connelly H, Hauser LJ, Wu LY, Liu XD, Yan TF, Leaphart A *et al*. 2004. Transcriptomic and proteomic characterization of the fur modulon in the metal-reducing bacterium *Shewanella oneidensis*. *J Bacteriol* **186:** (24) 8385.
Yao YF, Sturdevant DE, Otto M. 2005. Genomewide analysis of gene expression in *Staphylococcus epidermidis* biofilms: Insights into the pathophysiology of *S. epidermidis* biofilms and the role of phenol-soluble modulins in formation of biofilms. *J Infect Dis* **191:** (2) 289.
Zheng J, Zhao JF, Tao YZ, Wang JH, Liu YJ, Fu JJ, Jin Y, Gao P, Zhang JP, Bai YF *et al*. 2004. Isolation and analysis of water stress induced genes in maize seedlings by subtractive PCR and cDNA macroarray. *Plant Mol Biol* **55:** (6) 807.

### II Proteomics

Abbas A, Koc H, Liu F, Tien M. 2005. Fungal degradation of wood: Initial proteomic analysis of extracellular proteins of *Phanerochaete chrysosporium* grown on oak substrate. *Curr Genet* **47:** (1) 49.
Andersen JS, Lam YW, Leung AKL, Ong SE, Lyon CE, Lamond AI, Mann M. 2005. Nucleolar proteome dynamics. *Nature* **433:** (7021) 77.
Baginsky S, Siddique A, Gruissem W. 2004. Proteome analysis of tobacco bright yellow-2 (BY-2) cell culture plastids as a model for undifferentiated heterotrophic plastids. *J Proteome Res* **3:** (6) 1128.
Carter C, Pan SQ, Jan ZH, Avila EL, Girke T, Raikhel NV. 2004. The vegetable vacuole proteome of *Arabidopsis thaliana* reveals predicted and unexpected proteins. *Plant Cell* **16:** (12) 3285.
Chan SM, Ermann J, Su L, Fathman CG, Utz PJ. 2004. Protein microarrays for multiplex analysis of signal transduction pathways. *Nat Med* **10:** (12) 1390.
Goodchild A, Raftery M, Saunders NFW, Guilhaus M, Cavicchioli R. 2004. Biology of the cold adapted archaeon, *Methanococcoides burtonii* determined by proteomics using liquid chromatography-tandem mass spectrometry. *J Proteome Res* **3:** (6) 1164.
Hochholdinger F, Guo L, Schnable PS. 2004. Lateral roots affect the proteome of the primary root of maize *(Zea mays L.)*. *Plant Mol Biol* **56:** (3) 397.
Huang J, Zhu H, Haggarty SJ, Spring DR, Hwang H, Jin FL, Snyder M, Schreiber SL. 2004. Finding new components of the target of rapamycin (TOR) signaling network through chemical genetics and proteome chips. *Proc Natl Acad Sci U S A* **101:** (47) 16594.
Huber CG, Walcher W, Timperio AM, Troiani S, Porceddu A, Zolla L. 2004. Multidimensional proteomic analysis of photosynthetic membrane proteins by liquid extraction-ultracentrifugation-liquid chromatography-mass spectrometry. *Proteomics* **4:** (12) 3909.
Kim SJ, Jones RC, Cha CJ, Kweon O, Edmondson RD, Cerniglia CE. 2004. Identification of proteins induced by polycyclic aromatic hydrocarbon in *Mycobacterium vanbaalenii* PYR-1 using two-dimensional polyacrylamide gel electrophoresis and *de novo* sequencing methods. *Proteomics* **4:** (12) 3899.
Kobi D, Zugmeyer S, Potier S, Jaquet-Gutfreund L. 2004. Two-dimensional protein map of an "ale"-brewing yeast strain: Proteome dynamics during fermentation. *FEMS Yeast Res* **5:** (3) 213.
Levy F, Rabel D, Charlet M, Bulet P, Hoffmann JA, Ehret-Sabatier L.

Copyright © 2005 John Wiley & Sons, Ltd. 

2004. Peptidomic and proteomic analyses of the systemic immune response of *Drosophila*. *Biochimie* **86:** (9-10) 607.
McSheehy S, Kelly J, Tessier L, Mester Z. 2005. Identification of selenomethionine in selenized yeast using two-dimensional liquid chromatography-mass spectrometry based proteomic analysis. *Analyst* **130:** (1) 35.
Palmfeldt J, Levander F, Hahn-Hagerdal B, James P. 2004. Acidic proteome of growing and resting *Lactococcus lactis* metabolizing maltose. *Proteomics* **4:** (12) 3881.
Pendle AF, Clark GP, Boon R, Lewandowska D, Lam YW, Andersen J, Mann M, Lamond AI, Brown JWS, Shaw PJ. 2005. Proteomic analysis of the *Arabidopsis* nucleolus suggests novel nucleolar functions. *Mol Biol Cell* **16:** (1) 260.
Peng XX, Wu YJ, Chen JG, Wang SY. 2004. Proteomic approach to identify acute phase response-related proteins with low molecular weight in loach skin following injury. *Proteomics* **4:** (12) 3989.
Shishkin SS, Kovalyov LI, Kovalyova MA. 2004. Proteomic studies of human and other vertebrate muscle proteins. *Biochemistry* **69:** (11) 1283.
Sommer AP, Miyake N, Wickramasinghe NC, Narlikar JV, Al-Mufti S. 2004. Functions and possible provenance of primordial proteins. *J Proteome Res* **3:** (6) 1296.
Starck J, Kallenius G, Marklund BI, Andersson DI, Akerlund T. 2004. Comparative proteome analysis of *Mycobacterium tuberculosis* grown under aerobic and anaerobic conditions. *Microbiology* **150:** (11) 3821.
Wang Y, Lu G, Wong WPS, Vliegenthart JFG, Gerwig GJ, Lam KSL, Cooper GJS, Xu AM. 2004. Proteomic and functional characterization of endogenous adiponectin purified from fetal bovine serum. *Proteomics* **4:** (12) 3933.
Xu CX, Ren HX, Wang SY, Peng XX. 2004. Proteomic analysis of salt-sensitive outer membrane proteins of *Vibrio parahaemolyticus*. *Res Microbiol* **155:** (10) 835.
Zhang KL, Siino JS, Jones PR, Yau PM, Bradbury EM. 2004. A mass spectrometric "western blot" to evaluate the correlations between histone methylation and histone acetylation. *Proteomics* **4:** (12) 3765.

### I2 Protein structural genomics

Canaves JM, Page R, Wilson IA, Stevens RC. 2004. Protein biophysical properties that correlate with crystallization success in *Thermotoga maritima*: Maximum clustering strategy for structural genomics. *J Mol Biol* **344:** (4) 977.
Chandonia JM, Brenner SE. 2005. Implications of structural genomics target selection strategies: Pfam5000, whole genome, and random approaches. *Proteins* **58:** (1) 166.
Mikkat S, Koy C, Ulbrich M, Ringel B, Glocker MO. 2004. Mass spectrometric protein structure characterization reveals cause of migration differences of haptoglobin a chains in two-dimensional gel electrophoresis. *Proteomics* **4:** (12) 3921.
Quevillon-Cheruel S, Dominique L, Leulliot N, Graille M, Poupon A, De La Sierra-Gallay IL, Cong-Zhao Z, Collinet B, Janin J, Van Tilbeurgh H. 2004. The Paris-Sud yeast structural genomics pilot-project: From structure to function. *Biochimie* **86:** (9-10) 617.

### I4 Genomic approaches to development

Adjaye J. 2005. Whole-genome approaches for large-scale gene identification and expression analysis in mammalian preimplantation embryos. *Reprod Fertil Dev* **17:** (1-2) 37.
Bobek J, Halada P, Angelis J, Vohradsky J, Mikulik K. 2004. Activation and expression of proteins during synchronous germination of aerial spores of *Streptomyces granaticolori*. *Proteomics* **4:** (12) 3864.
Egertsdotter U, Van Zyl LM, MacKay J, Peter G, Kirst M, Clark C, Whetten R, Sederoff R. 2004. Gene expression during formation of earlywood and latewood in loblolly pine: Expression profiles of 350 genes. *Plant Biol* **6:** (6) 654.
Elliott ST, Crider DG, Garham CP, Boheler KR, Van Eyk JE. 2004. Two-dimensional gel electrophoresis database of cultured murine R1 embryonic cells. *Proteomics* **4:** (12) 3813.
Hall N, Karras M, Raine JD, Carlton JM, Kooij TWA, Berriman M, Florens L, Janssen CS, Pain A, Christophides GK *et al*. 2005. A comprehensive survey of the *Plasmodium* life cycle by genomic transcriptomic, and proetomic analyses. *Science* **307:** (5706) 82.
Jensen P, Magdaleno S, Lehman KM, Rice DS, LaVallie ER, Collins-Racie L, McCoy JM, Curran T. 2004. A neurogenomics approach to gene expression analysis in the developing brain. *Mol Brain Res* **132:** (2) 116.
Karlsson C, Korayem AM, Scherfer C, Loseva O, Dushay MS, Theopold U. 2004. Proteomic analysis of the *Drosophila* larval hemolymph clot. *J Biol Chem* **279:** (50) 52033.
Kezele PR, Ague JM, Nilsson E, Skinner MK. 2005. Alterations in the ovarian transcriptome during primordial follicle assembly and development. *Biol Reprod* **72:** (1) 241.
Lee GW, Yoon HC, Byun SY. 2004. Inhibitory effect of *Eucommia ulmoides* Oliver on adipogenic differentiation through proteome analysis. *Enzyme Microb Technol* **35:** (6-7) 632.
Li-Pook-Than J, Carrillo C, Bonen L. 2004. Variation in mitochondrial transcript profiles of protein-coding genes during early germination and seeding development in wheat. *Curr Genet* **46:** (6) 374.
Robson P. 2004. The maturing of the human embryonic stem cell transcriptome profile. *Trends Biotechnol* **22:** (12) 609.
Sirard MA, Dufort I, Vallee M, Massicotte L, Gravel C, Reghenas H, Watson AJ, King WA, Robert C. 2005. Potential and limitations of bovine-specific arrays for the analysis of mRNA levels in early development: Preliminary analysis using a bovine embryonic array. *Reprod Fertil Dev* **17:** (1-2) 47.

### I5 Technological advances

Barrett MT, Scheffer A, Ben-Dor A, Sampas N, Lipson D, Kincaid R, Tsang P, Curry B, Baird K, Meltzer PS *et al*. 2004. Comparative genomic hybridization using oligonucleotide microarrays and total genomic DNA. *Proc Natl Acad Sci U S A* **101:** (51) 17765.
Gauthier DJ, Gibbs BF, Rabah N, Lazure C. 2004. Utilization of a new biotinylation reagent in the development of a nondiscriminatory investigative approach for the study of cell surface proteins. *Proteomics* **4:** (12) 3783.
Hsu JL, Chou MK, Liang SS, Huang SY, Wu CJ, Shi FK, Chen SH. 2004. Photopolymerized microtips for sample preparation in proteomic analysis. *Electrophoresis* **25:** (21-22) 3840.
Le Bihan T, Robinson MD, Stewart II, Figeys D. 2004. Definition and characterization of a "trypsinosome" from specific peptide characteristics by nano-HPLC-MS/MS and *in silico* analysis of complex protein mixtures. *J Proteome Res* **3:** (6) 1138.
Lefler DM, Pafford RG, Black NA, Raymond JR, Arthur JM. 2004. Identification of proteins in slow continuous ultrafiltrate by reversed-phase chromatography and proteomics. *J Proteome Res* **3:** (6) 1254.
Moore LL, Fulton AM, Harrison ML, Geahlen RL. 2004. Anti-sulfonylbenzoate antibodies as a tool for the detection of nucleotide-binding proteins for functional proteomics. *J Proteome Res* **3:** (6) 1184.
Pan Q, Shai O, Misquitta C, Zhang W, Saltzman AL, Mohammad N, Babak T, Siu H, Hughes TR, Morris QD *et al*. 2004. Revealing global regulatory features of mammalian alternative splicing using a quantitative microarray platform. *Mol Cell* **16:** (6) 929.
Rahbar AM, Fenselau C. 2004. Integration of Jacobson's pellicle method into proteomic strategies for plasma membrane proteins. *J Proteome Res* **3:** (6) 1267.
Sethuraman M, McComb M, Huang H, Huang S, Heibeck T, Costello C, Cohen R. 2004. Isotope-coded affinity tag (ICAT) approach to redox proteomics: Identification and quantitation of oxidant-sensitive cysteine thiols in complex protein mixtures. *J Proteome Res* **3:** (6) 1228.
Thompson M, Cheran LE, Zhang M, Chacko M, Huo H, Sadeghi S. 2005. Label-free detection of nucleic acid and protein microarrays by scanning Kelvin nanoprobe. *Biosens Bioelectron* **20:** (8) 1471.
Vasilescu J, Gou XC, Kast J. 2004. Identification of protein-protein interactions using *in vivo* cross-linking and mass spectrometry. *Proteomics* **4:** (12) 3845.
Wu HL, Tian YP, Liu BH, Lu HJ, Wang XY, Zhai JJ, Jin H, Yang PY, Xu YM, Wang HH. 2004. Titania and alumina sol-gel-derived microfluidics enzymatic-reactors for peptide mapping: Design, characterization, and performance. *J Proteome Res* **3:** (6) 1201.
Zischka H, Gloeckner CJ, Klein C, Willmann S, Lange MSE, Ueffing M. 2004. Improved mass spectrometric identification of gel-separated hydrophobic membrane proteins after sodium dodecyl sulfate removal by ion-pair extraction. *Proteomics* **4:** (12) 3776.

Copyright © 2005 John Wiley & Sons, Ltd. 

### I6 Bioinformatics

Accardo MC, Giordano E, Riccardo S, Digilio FA, Iazzetti G, Calogero RA, Furia M. 2004. A computational search for box C/D snoRNA genes in the *Drosophila melanogaster* genome. *Bioinformatics* **20:** (18) 3293.

Anderle M, Roy S, Lin H, Becker C, Joho K. 2004. Quantifying reproducibility for differential proteomics: Noise analysis for protein liquid chromatography-mass spectrometry of human serum. *Bioinformatics* **20:** (18) 3575.

Antonov AV, Tetko IV, Prokopenko VV, Kosykh D, Mewes HW. 2004. A web portal for classification of expression data using maximal margin linear programming. *Bioinformatics* **20:** (17) 3284.

Bae K, Mallick BK. 2004. Gene selection using a two-level hierarchical Bayesian model. *Bioinformatics* **20:** (18) 3423.

Baird D, Johnstone P, Wilson T. 2004. Normalization of microarray data using a spatial mixed model analysis which includes splines. *Bioinformatics* **20:** (17) 3196.

Balasubramanian R, LaFramboise T, Scholtens D, Gentleman R. 2004. A graphic-theoretic approach to testing associations between disparate sources of functional genomics data. *Bioinformatics* **20:** (18) 3353.

Bhattacharjee M, Pritchard CC, Nelson PS, Arjas E. 2004. Bayesian integrated functional analysis of microarray data. *Bioinformatics* **20:** (17) 2943.

Bilbao-Castro JR, Sorzano COS, Garcia I, Fernandez JJ. 2004. Phan3D: Design of biological phantoms in 3D electron microscopy. *Bioinformatics* **20:** (17) 3286.

Blinov ML, Faeder JR, Goldstein B, Hlavacek WS. 2004. BioNetGen: Software for rule-based modeling of signal transduction based on the interactions of molecular domains. *Bioinformatics* **20:** (17) 3289.

Boyle EI, Weng SA, Gollub J, Jin H, Botstein D, Cherry JM, Sherlock G. 2004. GO::TermFinder - Open source software for accessing Gene Ontology information and finding significantly enriched Gene Ontology terms associated with a list of genes. *Bioinformatics* **20:** (18) 3710.

Buehler EC, Sachs JR, Shao K, Bagchi A, Ungar LH. 2004. The CRASSS plug-in for integrating annotation data with hierarchical clustering results. *Bioinformatics* **20:** (17) 3266.

Buisine N, Chalmers R. 2004. yBlast, a graphical front end for the standalone BLAST suite. *Biotechniques* **37:** (6) 987.

Chang JS, Van Remmen H, Ward WF, Regnier FE, Richardson A, Cornell J. 2004. Processing of data generated by 2-dimensional gel electrophoresis for statistical analysis: Missing data, normalization, and statistics. *J Proteome Res* **3:** (6) 1210.

Chen Y, Xu D. 2004. Global protein function annotation through mining genome-scale data in yeast *Saccharomyces cerevisiae*. *Nucleic Acids Res* **32:** (21) 6414.

Chuang TJ, Chen FC, Chou MY. 2004. A comparative method for identification of gene structures and alternatively spliced variants. *Bioinformatics* **20:** (17) 3064.

Craig R, Cortens JP, Beavis RC. 2004. Open source system for analyzing, validating, and storing protein identification data. *J Proteome Res* **3:** (6) 1234.

De la Fuente A, Bing N, Hoeschele I, Mendes P. 2004. Discovery of meaningful associations in genomic data using partial correlation coefficients. *Bioinformatics* **20:** (18) 3565.

Dettling M. 2004. BagBoosting for tumor classification with gene expression data. *Bioinformatics* **20:** (18) 3583.

Ding L, Sabo A, Berkowicz N, Meyer RR, Shotland Y, Johnson MR, Peoin KH, Wilson RK, Spieth J. 2004. EAnnot: A genome annotation tool using experimental evidence. *Genome Res* **14:** (12) 2503.

Dingare S, Nissim M, Finkel J, Manning C, Grover C. 2005. A system for identifying named entities in biomedical text: How results from two evaluations reflect on both the system and the evaluations (Conference Paper). *Comp Funct Genom* **6:** (1-2) 77.

Dowsey AW, Dunn MJ, Yang GZ. 2004. ProteomeGRID: Towards a high-throughput proteomics pipeline through opportunistic cluster image computing for two-dimensional gel electrophoresis. *Proteomics* **4:** (12) 3800.

Dragomir A, Mavroudi S, Bezerianos A. 2004. SOM-based class discovery exploring the ICA-reduced features of microarray expression profiles. *Comp Funct Genom* **5:** (8) 596.

Durinck S, Allemeersch J, Carey VJ, Moreau Y, De Moor B. 2004. Importing MAGE-ML format microarray data into BioConductor. *Bioinformatics* **20:** (18) 3641.

Eilbeck K, Lewis SE. 2004. Sequence Ontology Annotation Guide (Conference Paper). *Comp Funct Genom* **5:** (8) 642.

Feng WS, Wang LS, Zhu DM. 2004. CTRD: A fast applet for computing signed translocation distance between genomes. *Bioinformatics* **20:** (17) 3256.

Finocchiaro G, Parise P, Minardi SP, Alcalay M, Muller H. 2004. GenePicker: Replicate analysis of Affymetrix gene expression microarrays. *Bioinformatics* **20:** (18) 3670.

Fragoso G, De Coronado S, Haber M, Hartel F, Wright L. 2004. Overview and utilization of the NCI Thesaurus (Conference Paper). *Comp Funct Genom* **5:** (8) 648.

Fugsang A. 2004. Bias explorer: Measurements of compositional bias in EMBL and GenBank sequence files. *Antonie van Leeuwenhoek* **86:** (4) 313.

Gilbert D, Ugawa Y, Buchhorn M, Wee TT, Mizushima A, Kim H, Chon K, Weon S, Ma JC, Ichiyanagi Y *et al*. 2004. Bio-Mirror project for public bio-data distribution. *Bioinformatics* **20:** (17) 3238.

Girolami M, Breitling R. 2004. Biologically valid linear factor models of gene expression. *Bioinformatics* **20:** (17) 3021.

Gisel A, Panetta M, Grillo G, Licciulli VF, Liuni S, Saccone C, Pesole G. 2004. DNAfan: A software tool for automated extraction and analysis of user-defined sequence regions. *Bioinformatics* **20:** (18) 3676.

Graham WV, Tcheng DK, Shirk AL, Attene-Ramos MS, Welge ME, Gaskins HR. 2004. Phylomat: An automated protein motif analysis tool for phylogenomics. *J Proteome Res* **3:** (6) 1289.

Gustafsson JS, Ceasar R, Glasbey CA, Blomberg A, Rudemo M. 2004. Statistical exploration of variation in quantitative two-dimensional gel electrophoresis. *Proteomics* **4:** (12) 3791.

Haas BJ, Delcher AL, Wortman JR, Salzberg SL. 2004. DAGchainer: A tool for mining segmental genome duplications and synteny. *Bioinformatics* **20:** (18) 3643.

Hallin PF, Ussery DW. 2004. CBS Genome Atlas Database: A dynamic storage for bioinformatic results and sequence data. *Bioinformatics* **20:** (18) 3682.

Harkema H, Roberts I, Gaizauskas R, Hepple M. 2005. A web service for biomedical term look-up (Conference Paper). *Comp Funct Genom* **6:** (1-2) 86.

Haverty PM, Hsiao LL, Gullans SR, Hansen U, Weng ZP. 2004. Limited agreement among three global gene expression methods highlights the requirement for non-global validation. *Bioinformatics* **20:** (18) 3431.

He WQ. 2004. A spline function approach for detecting differentially expressed genes in microarray data analysis. *Bioinformatics* **20:** (17) 2954.

Hitte C, Derrien T, Andre C, Ostrander EA, Galibert F. 2004. CRH_Server: An online comparative and radiation hybrid mapping server for the canine genome. *Bioinformatics* **20:** (18) 3665.

Hsiao A, Worrall DS, Olefsky JM, Subramaniam S. 2004. Variance-modeled posterior inference of microarray data: Detecting gene-expression changes in 3T3-L1 adipocytes. *Bioinformatics* **20:** (17) 3108.

Huang TW, Tien AC, Lee YCG, Huang WS, Lee YCG, Peng CL, Tseng HH, Kao CY, Huang CYF. 2004. POINT: A database for the prediction of protein-protein interactions based on the orthologous interactome. *Bioinformatics* **20:** (17) 3273.

Hupe P, Stransky N, Thiery JP, Radvanyi F, Barillot E. 2004. Analysis of array CGH data: From signal ratio to gain and loss of DNA regions. *Bioinformatics* **20:** (18) 3413.

Jong K, Marchiori E, Meijer G, Van der Vaart A, Yistra B. 2004. Breakpoint identification and smoothing of array comparative genomic hybridization data. *Bioinformatics* **20:** (18) 3636.

Kaluszka A, Gibas C. 2004. Interactive gene-order comparison for multiple small genomes. *Bioinformatics* **20:** (18) 3662.

Kemmer D, Faxen M, Hodges E, Lim J, Herzog E, Ljungstrom E, Lundmark A, Olsen MK, Podowski R, Sonnhammer ELL, Nilsson P, Reimers M, Lenhard B, Roberds SL, Wahlestedt C, Hoog C, Agarwal P, Wasserman WW. 2004. Exploring the foundation of genomics: A Northern blot reference set for the comparative analysis of transcript profiling technologies. *Comp Funct Genom* **5:** (8) 584.

Knowlton N, Dozmorov IM, Centola M. 2004. Microarray Data Analysis Toolbox (MDAT): For normalization, adjustment and analysis of gene expression data. *Bioinformatics* **20:** (18) 3687.

Copyright © 2005 John Wiley & Sons, Ltd.

Lai YL, Wu BL, Chen L, Zhao HY. 2004. A statistical method for identifying differential gene-gene co-expression patterns. *Bioinformatics* **20:** (17) 3146.
Lee S, Cho MK, Jung JW, Kim JH, Lee W. 2004. Exploring protein fold space by secondary structure prediction using data distribution method on Grid platform. *Bioinformatics* **20:** (18) 3500.
Leinonen R, Diez FG, Binns D, Fleischmann W, Lopez R, Apweiler R. 2004. UniProt archive. *Bioinformatics* **20:** (17) 3236.
Li LX, Li HZ. 2004. Dimension reduction methods for microarrays with application to censored survival data. *Bioinformatics* **20:** (18) 3406.
Li X, Rao SQ, Zhang TW, Guo Z, Zhang QP, Moser KL, Topol EJ. 2004. An ensemble method for gene discovery based on DNA microarray data. *Sci China C Life Sci* **47:** (5) 396.
Liao BY, Chang YJ, Ho JM, Hwang MJ. 2004. The UniMarker (UM) method for synteny mapping of large genomes. *Bioinformatics* **20:** (17) 3156.
Linder R, Dew D, Sudhoff H, Theegarten D, Remberger K, Poppl SJ, Wagner M. 2004. The subsequent artificial neural network' (SANN) approach might bring more classificatory power to ANN-based DNA microarray analyses. *Bioinformatics* **20:** (18) 3544.
Liu SL, Tinker NA, Molnar SJ, Mather DE. 2004. EC_oligos: Automated and whole-genome primer design for exons within one or between two genomes. *Bioinformatics* **20:** (18) 3668.
Mani I, Hu Z, Jang SB, Samuel K, Krause M, Phillips J, Wu CH. 2005. Protein name tagging guidelines: Lessons learned (Conference Paper). *Comp Funct Genom* **6:** (1-2) 72.
Masseroli M, Stella A, Meani N, Alcalay M, Pinciroli F. 2004. MyWEST: My Web extraction software tool for effective mining of annotations from web-based databanks. *Bioinformatics* **20:** (18) 3326.
McDonald DM, Chen HC, Su H, Marshall BB. 2004. Extracting gene pathway relations using a hybrid grammar: The Arizona Relation Parser. *Bioinformatics* **20:** (18) 3370.
McDonald RT, Winters RS, Mandel M, Jin Y, White PS, Pereira F. 2004. An entity tagger for recognizing acquired genomic variations in cancer literature. *Bioinformatics* **20:** (17) 3249.
McKinney JL, Murdoch DJ, Wang J, Robinson J, Biltcliffe C, Khan HMR, Walker PM, Savage J, Skerjanc I, Hegele RA. 2004. Venn analysis as part of a bioinformatic approach to prioritize expressed sequence tags from cardiac libraries. *Clin Biochem* **37:** (11) 953.
Milward D, Bjareland M, Hayes W, Maxwell M, Oberg L, Tilford N, Thomas J, Hale R, Knight S, Barnes J. 2005. Ontology-based interactive information extraction from scientific abstracts (Conference Paper). *Comp Funct Genom* **6:** (1-2) 67.
Myers CL, Dunham MJ, Kung SY, Troyanskaya OG. 2004. Accurate detection of aneuploidies in array CGH and gene expression microarray data. *Bioinformatics* **20:** (18) 3533.
Neuhauser M, Senske R. 2004. The Baumgartner-Weiss-Schindler test for the detection of differentially expressed genes in replicated microarray experiments. *Bioinformatics* **20:** (18) 3553.
Niu TH, Hu ZJ. 2004. SNPicker: A graphical tool for primer picking in designing mutagenic endonuclease restriction assays. *Bioinformatics* **20:** (17) 3263.
Oinn T, Addis M, Ferris J, Marvin D, Senger M, Greenwood M, Carver T, Glover K, Pocock MR, Wipat A *et al*. 2004. Taverna: A tool for the composition and enactment of bioinformatics workflows. *Bioinformatics* **20:** (17) 3045.
Pham TD, Zuegg J. 2004. A probabilistic measure for alignment-free sequence comparison. *Bioinformatics* **20:** (18) 3455.
Pochet N, De Smet F, Suykens JAK, De Moor BLR. 2004. Systematic benchmarking of microarray data classification: Assessing the role of non-linearity and dimensionality reduction. *Bioinformatics* **20:** (17) 3185.
Pournara I, Wernisch L. 2004. Reconstruction of gene networks using Bayesian learning and manipulation experiments. *Bioinformatics* **20:** (17) 2934.
Rebeiz M, Posakony JW. 2004. GenePalette: A universal software tool for genome sequence visualization and analysis. *Dev Biol* **271:** (2) 431.
Roberts M, Hayes W, Hunt BR, Mount SM, Yorke JA. 2004. Reducing storage requirements for biological sequence comparison. *Bioinformatics* **20:** (18) 3363.
Rosenfeld JA, Sarkar IN, Planet PJ, Figurski DH, DeSalle R. 2004. ORFcurator: Molecular curation of genes and gene clusters in prokaryotic organisms. *Bioinformatics* **20:** (18) 3462.
Saldanha AJ. 2004. Java Treeview-extensible visualization of microarray data. *Bioinformatics* **20:** (17) 3246.
Samuelsson J, Dalevi D, Levander F, Rognvaldsson T. 2004. Modular, scriptable and automated analysis tools for high-throughput peptide mass fingerprinting. *Bioinformatics* **20:** (18) 3628.
Satten GA, Datta S, Moura H, Woolfitt AR, Carvalho MD, Carlone GM, De BK, Pavlopoulos A, Barr JR. 2004. Standardization and denoising algorithms for mass spectra to classify whole-organism bacterial specimens. *Bioinformatics* **20:** (17) 3128.
Sawkins MC, Farmer AD, Hoisington D, Sullivan J, Tolopko A, Jiang Z, Ribaut JM. 2004. Comparative Map and Trait Viewer (CMTV): An integrated bioinformatic tool to construct consensus maps and compare QTL and functional genomics data across genomes and experiments. *Plant Mol Biol* **56:** (3) 465.
Sese J, Kurokawa Y, Monden M, Kato K, Morishita S. 2004. Constrained clusters of gene expression profiles with pathological features. *Bioinformatics* **20:** (17) 3137.
Shaw OJ, Harwood C, Steggles LJ, Wipat A. 2004. SARGE: A tool for creation of putative genetic networks. *Bioinformatics* **20:** (18) 3638.
Shih JH, Michalowska AM, Dobbin K, Ye YM, Qiu TH, Green JE. 2004. Effects of pooling mRNA in microarray class comparisons. *Bioinformatics* **20:** (18) 3318.
Shoop E, Casaes P, Onsongo G, Lesnett L, Petursdottir FO, Donkor EKY, Tkach D, Cosimini M. 2004. Data exploration tools for the Gene Ontology database. *Bioinformatics* **20:** (18) 3442.
Simpson CL, Hansen VK, Sham PC, Collins A, Powell JF, Al-Chalabi A. 2004. MaGIC: A program to generate targeted marker sets for genome-wide association studies. *Biotechniques* **37:** (6) 996.
Steinhauser D, Usadel B, Luedemann A, Thimm O, Kopka J. 2004. CSB.DB: A comprehensive systems-biology database. *Bioinformatics* **20:** (18) 3647.
Thomas R, Mehrotra S, Papoutsakis ET, Hatzimanikatis V. 2004. A model-based optimization framework for the inference on gene regulatory networks from DNA array data. *Bioinformatics* **20:** (17) 3221.
Vaquerizas JM, Dopazo J, Diaz-Uriarte R. 2004. DNMAD: Web-based diagnosis and normalization for microarray data. *Bioinformatics* **20:** (18) 3656.
Verspoor K. 2005. Towards a semantic lexicon for biological language processing (Conference Paper). *Comp Funct Genom* **6:** (1-2) 61.
Wang J, Coombes KR, Highsmith WE, Keating MJ, Abruzzo LV. 2004. Differences in gene expression between B-cell chronic lymphocytic leukemia and normal B cells: A meta-analysis of three microarray studies. *Bioinformatics* **20:** (17) 3166.
Wang JPZ, Lindsay BG, Leebens-Mack J, Cui LY, Wall K, Miller WC, DePamphilis CW. 2004. EST clustering error evaluation and correction. *Bioinformatics* **20:** (17) 2973.
Wang Y, Leung FCC. 2004. DNA structure constraint is probably a fundamental factor inducing CpG deficiency in bacteria. *Bioinformatics* **20:** (18) 3336.
Wettenhall JM, Smyth GK. 2004. limmaGUI: A graphical user interface for linear modeling of microarray data. *Bioinformatics* **20:** (18) 3705.
Wisz MS, Suarez MK, Holmes MR, Giddings MC. 2004. GFSWeb: A web tool for genome-based identification of proteins from mass spectrometric samples. *J Proteome Res* **3:** (6) 1292.
Wu CC, Huang HC, Juan HF, Chen ST. 2004. GeneNetwork: An interactive tool for reconstruction of genetic networks using microarray data. *Bioinformatics* **20:** (18) 3691.
Wyman SK, Jansen RK, Boore JL. 2004. Automatic annotation of organellar genomes with DOGMA. *Bioinformatics* **20:** (17) 3252.
Xu XJ, Wang LS, Ding DF. 2004. Learning module networks from genome-wide location and expression data. *FEBS Lett* **578:** (3) 297.
Zbilut JP, Giuliani A, Colosimo A, Mitchell JC, Colafranceschi M, Marwan N, Webber CL, Uversky VN. 2004. Charge and hydrophobicity patterning along the sequence predicts the folding mechanism and aggregation of proteins: A computational approach. *J Proteome Res* **3:** (6) 1243.
Zheng CJ, Sun LZ, Han LY, Ji ZL, Chen X, Chen YZ. 2004. Drug ADME-associated protein database as a resource for facilitating pharmacogenomics research. *Drug Dev Res* **62:** (2) 134.
Zhu HQ, Hu GQ, Ouyang ZQ, Wang J, She ZS. 2004. Accuracy improvement for identifying translation initiation sites in microbial genomes. *Bioinformatics* **20:** (18) 3308.

Copyright © 2005 John Wiley & Sons, Ltd. 
